# Are you Discussing Dental Caries in Children with Current and Local References? - Letter to The Editor

**DOI:** 10.31729/jnma.3631

**Published:** 2018-10-31

**Authors:** Martin Hofmeister

**Affiliations:** 1 Consumer Centre of the German Federal State of Bavaria, Department of Food and Nutrition, Mozartsraß e 9, D-80336, Munich, Germany

**Dear Editor**,

I congratulate the talented team of Dikshit et al^1^ for their interesting clinical study on the association of body mass index (BMI) with dental caries among Nepalese children, published in a recent issue of the Journal of Nepal Medical Association (JNMA, Mar–Apr, 2018).^1^ There are some aspects worth mentioning.

As a nutrition scientist, I wonder why the authors have highlighted in the discussion section that “... the caries prevalence in children has not shown a significant decline”.^1^ This statement is not correct. For example, a comprehensive review conducted by Frencken et al (published in March 2017) found sufficient evidence that the prevalence and severity of cavitated dentine carious lesions among 5- and 12-year-olds has decreased over the last three decades.^2^ Other published data analyses from 2016/2017 have also shown a significant decline in the prevalence and severity of dental caries in children in countries such as Brazil, China, Korea, Thailand, or United States.^3–7^

The authors observed that the prevalence of dental caries of the study participants was 90%. Other investigations with children in Nepal resulted in lower prevalence data (Table 1).

**Table 1.** Prevalence of dental caries in Nepalese children by districts.

District	Age group	Percent of dental caries	Sample size	Reference
**Pokhara**	5–7 years	20.7	3174	Adhikari et al (2012)^8^
	8–10 years	48.2		
	11–14 years	52.4		
**Chitwan**	5–6 years	52.0	361	Dixit et al (2013)^9^
	12–13 years	41.0		
**Dhankuta and Sunsari**	5–12 years	56.0	616	Bhagat et al (2014)^10^
**Kathmandu**	12–15 years	42.6	366	Shrestha et al (2014)^11^
**Kathmandu**	12–15 years	58.3	252	Khanal et al (2014)^12^
**Nawalparasi**	5–6 years	64.4	826	Thapa et al (2015)^13^
	12–13 years	42.2		

I think in the discussion section the findings should also be compared with existing local literature. For example, in 2016, the relationship of BMI with caries experience (decayed, missing and filled teeth - DMFT) was evaluated at 208 Nepalese children of age three years to six years, who visited the Pediatric Dentistry department of Dhulikhel Hospital, Kavre. Similar to the authors, Upadhyay et al also found no significant association between DMFT and BMI z-scores in the younger studied population.^14^

I fully agree with the authors that there are contradictory results and further local research is needed to examine the relationship between body composition and dental caries among children. The result of a recent meta-analysis of Sun Yat-sen University (Guangzhou, China) is also interesting for this BMI association: Overweight and obese children had significantly more dental caries in their primary as well as permanent teeth in high-income countries, but not in low- and middle-income countries.^15^ Apart from the fact that more confounding factors need to be controlled in addition to standardized measurements in future “oral health” data analyses, the question arises whether other anthropometric parameters such as waist circumference or waist-to-hip ratio are better suited than BMI, in particular for long term associations?^16, 17^

Despite the remarkably nutritional achievements of recent years in Nepal,^18^ the authors rightly point out that undernutrition among children is still a major public health problem. In the introduction, a study by Ghosh et al^19^ is cited, which already recorded its high prevalence data in 2007 (44.5% stunting and 49.3% underweight). According to the Nepal Demographic and Health Survey, also mentioned by the authors, the prevalence of stunting and underweight among children under five years has fallen in the last 10 years: Stunting 49% in 2006 to 36% in 2016, and underweight 39% in 2006 to 27% in 2016.^20^ Overall, I think that the good examination by Dikshit et al^1^ can be strengthened by additional discussion.

## Conflict of Interest

**None.**
